# Cooperative and Independent Functions of the *miR-23a~27a~24-2* Cluster in Bovine Adipocyte Adipogenesis

**DOI:** 10.3390/ijms19123957

**Published:** 2018-12-09

**Authors:** Yaning Wang, Yingying Zhang, Xiaotong Su, Hongbao Wang, Wucai Yang, Linsen Zan

**Affiliations:** 1 College of Animal Science and Technology, Northwest A&F University, Yangling 712100, China; wangyn1992@outlook.com (Y.W.); xiaotongsu86@gmail.com (X.S.); wanghongbao@nwsuaf.edu.cn (H.W.); yangwucai111@163.com (W.Y.); 2 Animal Husbandry and Veterinary Research Institute, Shanghai Academy of Agricultural Sciences, Shanghai 201106, China; zyy686868@163.com; 3 National Beef Cattle Improvement Center in China, Yangling 712100, China

**Keywords:** *miR-23a~27a~24-2* cluster, bovine, adipocyte, adipogenesis

## Abstract

The *miR-23a~27a~24-2* cluster is an important regulator in cell metabolism. However, the cooperative and independent functions of this cluster in bovine adipocyte adipogenesis have not been elucidated. In this study, we found that expression of the *miR-23a~27a~24-2* cluster was induced during adipogenesis and this cluster acted as a negative regulator of adipogenesis. *miR-27a* and *miR-24-2* were shown to inhibit adipogenesis by directly targeting glycerol-3-phosphate acyltransferase, mitochondrial (*GPAM*) and diacylglycerol O-acyltransferase 2 (*DGAT2*), both of which promoted adipogenesis. Meanwhile, *miR-23a* and *miR-24-2* were shown to target decorin (*DCN*), glucose-6-phosphate dehydrogenase (*G6PD*), and lipoprotein lipase (*LPL*), all of which repressed adipogenesis in this study. Thus, the *miR-23a~27a~24-2* cluster exhibits a non-canonical regulatory role in bovine adipocyte adipogenesis. To determine how the *miR-23a~27a~24-2* cluster inhibits adipogenesis while targeting anti-adipogenic genes, we identified another target gene, fibroblast growth factor 11 (*FGF11*), a positive regulator of adipogenesis, that was commonly targeted by the entire *miR-23a~27a~24-2* cluster. Our findings suggest that the *miR-23a~27a~24-2* cluster fine-tunes the regulation of adipogenesis by targeting two types of genes with pro- or anti-adipogenic effects. This balanced regulatory role of *miR-23a~27a~24-2* cluster finally repressed adipogenesis.

## 1. Introduction

Fat deposition is highly correlated with beef carcass grading and characteristics that contribute to meat quality such as juiciness, tenderness, and flavor. Therefore, it is essential to advance our understanding of the molecular mechanisms that affect fat deposition so that genomic selection can be used to produce high-grade beef with consistent quality. Adipose tissue is a remarkably complex organ that is involved in multiple physiological and pathological processes [1]. Adipogenesis of progenitor cells into mature adipocytes is tightly controlled by various factors [2,3]. Among these factors, miRNAs have been reported to have a close relationship with fat deposits and meat quality [4,5]. However, the exact roles of many miRNAs in adipogenesis have not yet been determined.

Mature miRNAs are small (approximately 22 nt), single-stranded non-cording RNAs that mainly repress gene expression at the post-transcriptional level by binding to target mRNAs [6]. During adipose tissue development and other biological processes, miRNAs mainly function individually or as clusters [7,8]. Generally, clustered miRNAs are expressed from the same transcript [9], and the host gene encoding these miRNAs possesses an intact gene structure [10]. The primary miRNA transcripts also contain a cap structure and a poly A tail like protein coding genes [10]. A previous study found that 37% of the known human miRNAs are located in clusters, and most of the clusters show a high degree of evolutionary conservation [11]. Usually, a miRNA cluster contains several to dozens of miRNAs, and the clustered miRNAs have similar but also individual functions [12].

The *miR-23a~27a~24-2* cluster, which is expressed from a single primary transcript, is involved in various physiological and pathological processes [12]. However, the expression patterns and functions of the miRNAs in this cluster are not always the same and are even opposite depending on the cell type and biological process [12]. Studies have shown that the *miR-23a~27a~24-2* cluster regulates endothelial cell apoptosis [13], osteoblast differentiation [14,15], neuronal apoptosis [16], and erythropoiesis [17]. Recent studies in humans have also indicated that the *miR-23a~27a~24-2* cluster may also significantly affect adipogenesis because many of their predicted target genes are involved in multiple signaling pathways related to lipid metabolism [12]. During the differentiation of bovine adipocyte progenitor cells and 3T3-L1 cells, *miR-23a* serves as a negative regulator [18,19]. *miR-27a* represses 3T3-L1 preadipocyte differentiation by directly targeting peroxisome proliferator activated receptor gamma (*PPARγ*) [20]. *miR-27a* is also involved in brown adipogenesis [21], porcine preadipocyte differentiation [22], and high-fat diet induced insulin resistance [23]. Based on these reports, we hypothesize that the functions of the miRNAs in this cluster are not exactly the same and that they have the crucial cooperative regulatory roles in the process of bovine preadipocyte differentiation. Therefore, it is essential to elucidate the molecular mechanisms by which the *miR-23a~27a~24-2* cluster affects fat deposition so that this knowledge can be applied to genomic selection to produce high-grade beef.

In the process of preadipocyte differentiation, fatty acids, and lipid synthesis pathways, such as oxidative phosphorylation, pentose phosphate pathway, and triacylglycerol (TAG) synthesis pathway, play essential roles [24,25,26]. In these cellular bioprocesses, adenosine triphosphate synthase peripheral stalk-membrane subunit b (ATP5F1) is important for oxidative phosphorylation [25], G6PD is essential for pentose phosphate pathway [27], and GPAM, DGAT2, DCN, and LPL are the key regulators for TAG synthesis [28,29,30]. Although it is much clearer how these genes affect adipogenesis, the interactions between these pathways and miRNA networks are largely unknown.

The aim of this study was to identify the mechanisms of the *miR-23a~27a~24-2* cluster in regulating bovine adipocyte adipogenesis. Through computational analysis, we found that there might be post-transcriptional regulations between the miRNAs in the *miR-23a~27a~24-2* cluster and genes related to lipid synthesis. Consequently, we hypothesize that the *miR-23a~27a~24-2* cluster might influence adipogenesis through interactions with lipid synthesis-related pathways. It is intriguing that the newly identified cooperative and independent regulatory roles of the miRNAs in this cluster will help inform future studies of preadipocyte differentiation, which can be applied to enhance fat deposition and meat quality of beef.

## 2. Results

### 2.1. Isolation and Adipogenic Differentiation of Bovine Preadipocytes

**Figure 1:**
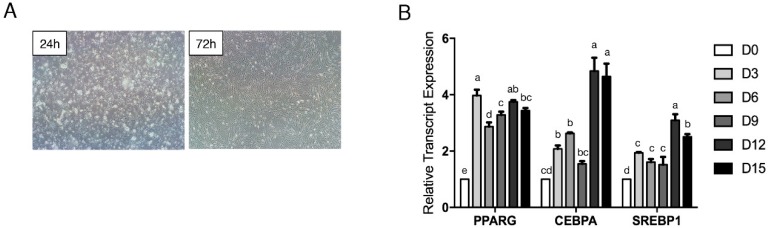
Isolation and adipogenic differentiation of bovine preadipocytes. (**A**) Initial isolation and cell culture of bovine primary preadipocytes at 24 and 72 h (magnification: 40×). (**B**) Time course of *PPARG*, *CEBPA*, and *SREBP1* expression during preadipocyte differentiation. Error bars represent SEM. Lowercases represent *p* < 0.05. Uppercases represent *p* < 0.01.

To establish a system to study the effects of miRNAs on the adipogenic differentiation of bovine preadipocytes, the adipogenic potential of preadipocytes isolated from perirenal adipose tissue was evaluated. As shown in Figure 1A, 24 h after isolation, cells started to adhere to the dishes and began to elongate. Seventy-two hours later, cells had grown to 70% confluence and were spindle-shaped (Figure 1A). When cells were 100% confluent, adipogenic differentiation of the preadipocytes was induced. The shape of the preadipocytes gradually changed from shuttle-shaped to oblate during adipogenesis. On the 15th day of differentiation, almost all cells were induced to mature adipocytes with strong adipogenic potential. The expression levels of the adipogenic marker genes *PPARG*, CCAAT enhancer binding protein alpha (*CEBPA*), and sterol regulatory element binding transcription factor 1 (*SREBP1*) were all obviously induced during differentiation (Figure 1B). These results indicate that the isolated bovine preadipocytes were suitable for subsequent experiments.

### 2.2. The miR-23a~27a~24-2 Cluster Represses Preadipocyte Differentiation

**Figure 2:**
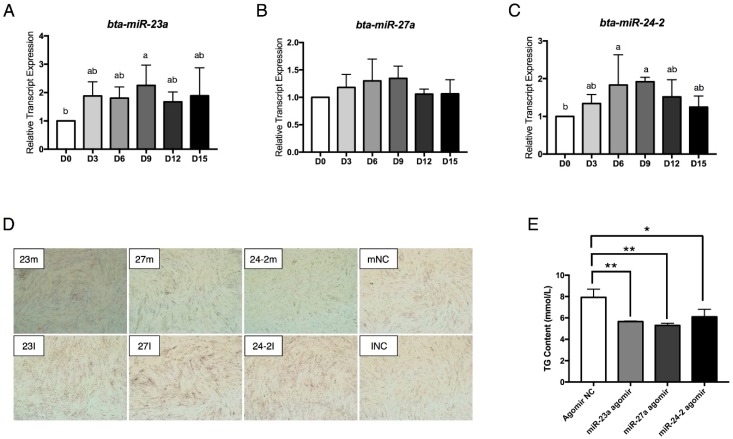
The *miR-23a~27a~24-2* cluster represses preadipocyte differentiation. (**A**–**C**) Time course of *miR-23a*, *miR-27a*, and *miR-24-2* expression during preadipocyte differentiation. (**D**) Oil red O staining of lipids in differentiated adipocytes transfected with *miR-23a* agomir (23m)/antagomir (23I), *miR-27a* agomir (27m)/antagomir (27I), *miR-24-2* agomir (24-2m)/antagomir (24-2I), negative control (NC) for agomir (mNC) and NC for antagomir (INC, magnification: 40×). (**E**) ELISA analysis of TG content in adipocytes transfected with Agomir NC, *miR-23a* agomir, *miR-27a* agomir and *miR-24-2* agomir. Error bars represent SEM. Lowercase represents *p* < 0.05. Uppercase represents *p* < 0.01. “*” represents *p* < 0.05; “**” represents *p* < 0.01.

To reveal the relationships between the *miR-23a~27a~24-2* cluster and adipogenesis, the time course of *miR-23a*, *miR-27a*, and *miR-24-2* expression from day 0 (D0) to D15 of differentiation were analyzed by performing quantitative real time PCR (qRT-PCR) (Figure 2A–C). *miR-23a* expression was induced starting on D3 and then remained stable (Figure 2A). *miR-24-2* expression was also induced from D3 to D9 and then the expression level gradually decreased (Figure 2C). 

To investigate the effects of *miR-23a*, *miR-27a*, and *miR-24-2* on preadipocyte differentiation, *miR-23a* agomir/antagomir, *miR-27a* agomir/antagomir, and *miR-24-2* agomir/antagomir were transfected individually into proliferating preadipocytes. When the cells were 100% confluent, the preadipocytes were induced to differentiation. The lipid droplets were observed by staining the cells with Oil Red O at D5 after adipogenic induction. As shown in Figure 2D, the *miR-23a*, *miR-27a*, and *miR-24-2* agomir repressed lipid droplet formation. In contrast, the antagomir promoted preadipocyte differentiation and increased lipid accumulation. Meanwhile, the total cytoplasm triglyceride (TG) content was also significantly repressed by the miRNA agomir (Figure 2E). These results indicate that the *miR-23a~27a~24-2* cluster is a negative regulator of bovine preadipocyte differentiation.

### 2.3. The miR-23a~27a~24-2 Cluster Negatively Regulates the Expression of ATP5F1, DCN, GPAM, DGAT2, G6PD, and LPL

**Figure 3:**
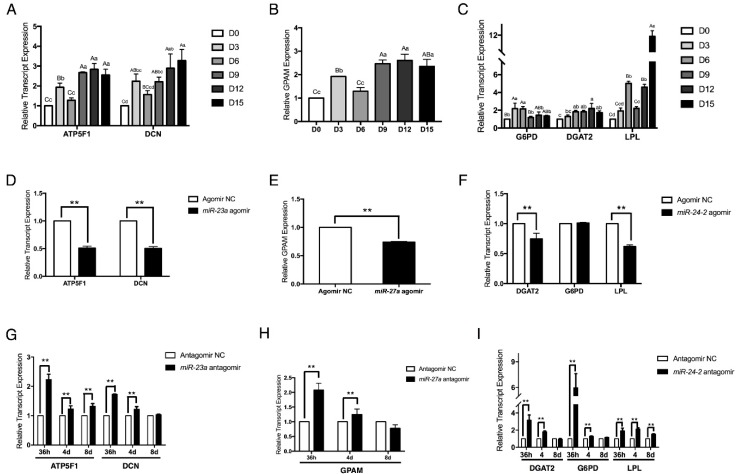
The *miR-23a~27a~24-2* cluster negatively regulates the expression of *ATP5F1*, *DCN*, *GPAM*, *DGAT2*, *G6PD*, and *LPL*. (**A**–**C**) Time course of *ATP5F1*, *DCN*, *GPAM*, *DGAT2*, *G6PD*, and *LPL* expression during preadipocyte differentiation. (**D**–**F**) mRNA expression of *ATP5F1*, *DCN*, *GPAM*, *DGAT2*, *G6PD*, and *LPL* in preadipocytes individually transfected with *miR-23a* agomir, *miR-27a* agomir, and *miR-24-2* agomir, followed by adipogenic differentiation at 36 h. (**G**–**I**) Transfection of *miR-23a* antagomir, *miR-27a* antagomir, and *miR-24-2* antagomir in preadipocytes, followed by adipogenic differentiation increased *ATP5F1*, *DCN*, *GPAM*, *DGAT2*, *G6PD*, and *LPL* expression from 36 h to 8 days. Error bars represent SEM. Lowercase represents *p* < 0.05. Uppercase represents *p* < 0.01. “**” represents *p* < 0.01.

By using TargetScan software (Release: 7.2: http://www.targetscan.org/vert_72/) and miRanda (http://www.microrna.org/microrna/home.do), six genes were identified as potential targets of the *miR-23a~27a~24-2* cluster: *ATP5F1*, *DCN* for *miR-23a*, *GPAM* for *miR-27a*, and *DGAT2*, *G6PD*, and *LPL* for *miR-24-2*. First, the expression patterns of the six genes were analyzed using qRT-PCR. As shown in Figure 3A–C, apart from *DGAT2*, whose expression was continuously induced from D0 to D12 and decreased at D15, gene expression levels of the other five genes first increased at D3 of differentiation, and then decreased at D6 to D9 before increasing again. These expression patterns are opposite to those of *miR-23a*, *miR-27a*, and *miR-24-2*, suggesting that these six genes might be regulated by the *miR-23a~27a~24-2* cluster. To verify this, the expression levels of the six genes were analyzed with adipocytes transfected with miRNA agomir or antagomir. Results showed that the *miR-23a* agomir strongly repressed *ATP5F1* and *DCN* expression (Figure 3D). In contrast, the *miR-23a* antagomir obviously increased the expression of these genes from 36 h to 8 days post transfection (Figure 3G). The expression of *GPAM* was also inhibited by the *miR-27a* agomir and induced by the *miR-27a* antagomir (Figure 3E,H). The *miR-24-2* agomir significantly reduced the expression of *DGAT2* and *LPL*, but had no effect on *G6PD* expression at 36 h post transfection (Figure 3F). By contrast, inhibition of *miR-24-2* by its antagomir strongly increased *DGAT2*, *G6PD*, and *LPL* expression; this increase was especially pronounced for *G6PD* at 36 h post transfection (Figure 3I). These results strongly suggest that *ATP5F1*, *DCN*, *GPAM*, *DGAT2*, *G6PD*, and *LPL* are targets of the *miR-23a~27a~24-2* cluster.

### 2.4. The miR-23a~27a~24-2 Cluster Directly Targets Two Types of Genes: GPAM and DGAT2 with Pro-Adipogenic Effects and DCN, G6PD, and LPL with Anti-Adipogenic Effects

**Figure 4:**
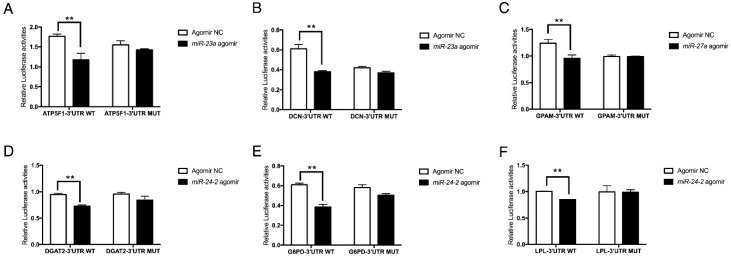
The *miR-23a~27a~24-2* cluster directly targets *ATP5F1*, *DCN*, *GPAM*, *DGAT2*, *G6PD*, and *LPL*. (**A**,**B**) Luciferase activity assay of *ATP5F1*-3’UTR and *DCN*-3’UTR targeted by *miR-23a*. (**C**) Luciferase activity assay of *GPAM*-3’UTR targeted by *miR-27a*. (**D**–**F**) Luciferase activity assay of *DGAT2*-3’UTR, *G6PD*-3’UTR, and *LPL*-3’UTR targeted by *miR-24-2*. Error bars represent SEM. “**” represents *p* < 0.01.

To investigate whether *ATP5F1*, *DCN*, *GPAM*, *DGAT2*, *G6PD*, and *LPL* were direct targets of the *miR-23a~27a~24-2* cluster, in vitro luciferase activity assays were performed. Wild type or mutant 3′UTR luciferase reporter vectors were co-transfected with miRNA agomir or the NC agomir in 293T cells. Results showed that *miR-23a* agomir significantly reduced the luciferase activities of the *ATP5F1* and *DCN* 3’UTR reporter vectors (Figure 4A,B), and the *miR-27a* agomir repressed the luciferase activity of the *GPAM* reporter vector (Figure 4C). The *miR-24-2* agomir clearly decreased the luciferase activity of the *DGAT2*, *G6PD*, and *LPL* reporter vectors (Figure 4D–F). The results from these luciferase activity assays confirmed that *ATP5F1*, *DCN*, *GPAM*, *DGAT2*, *G6PD*, and *LPL* are direct target genes of the *miR-23a~27a~24-2* cluster.

**Figure 5:**
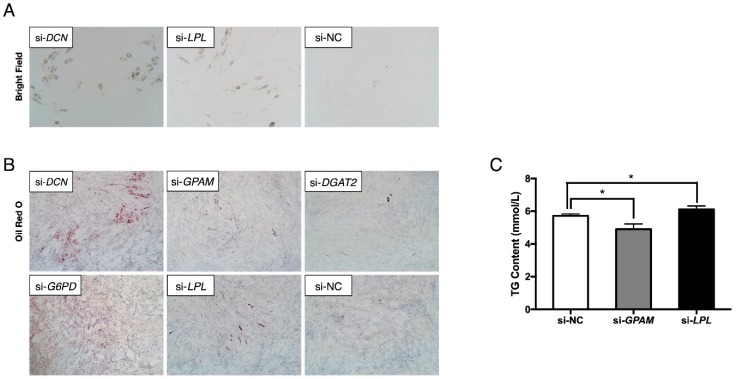
Interference effects of *DCN*, *GPAM*, *DGAT2*, *G6PD*, and *LPL* on adipocyte adipogenesis. (**A**) Bright field microscopy of lipid droplets in *DCN*- and *LPL*-interfered preadipocytes followed by differentiation at day 5 (magnification: 100×). As there were no obvious lipid droplets observed in the bright field microscopy when interfering *GPAM*, *DGAT2*, and *G6PD*, the data was not shown. (**B**–**C**) Oil Red O staining of lipids (magnification: 40×) and ELISA analysis of TG content showed that *DCN*, *G6PD*, and *LPL* were strongly anti-adipogenic while *GPAM* and *DGAT2* were pro-adipogenic in adipogenesis. Error bars represent SEM. “*” represents *p* < 0.05.

Most of the present published literatures reported that the effects of miRNA and their targets were opposite [14,31]. To investigate the roles of the six target genes on preadipocyte differentiation, interference assays were performed using siRNA. Due to the difficulty of designing an siRNA targeting *ATP5F1* mRNA, *ATP5F1* was not included in this study. Surprisingly, transfection of cells with siRNA targeting *DCN* or *LPL* significantly increased lipid content, as visualized by bright field microscopy (Figure 5A) and Oil Red O staining (Figure 5B). ELISA analysis of TG content also showed that inhibition of *LPL* expression increased adipocyte TG content (Figure 5C). Similarly, interference of *G6PD* obviously promoted adipogenesis, but inhibition of *DGAT2* reduced lipid content (Figure 5B). As it was not easy to recognize how *GPAM* affected adipogenesis using Oil Red O staining (Figure 5B), and the results of ELISA analysis showed that the interference of *GPAM* expression reduced adipocyte TG content. These results suggest that the five targets were sorted into two groups: *GPAM* and *DGAT2* with pro-adipogenic effects, and *DCN*, *G6PD*, and *LPL* with anti-adipogenic effects. However, it is not clear how the miRNA cluster acts as a negative regulator of adipogenesis when most of its target genes are anti-adipogenic.

### 2.5. Hypothesis: There are Other Pro-Adipogenic Target Genes that Contribute to the miR-23a~27a~24-2 Cluster-Regulated Adipogenesis

**Figure 6:**
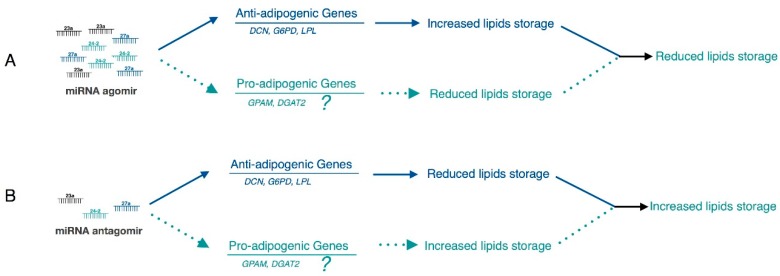
A hypothesis of the *miR-23a~27a~24-2* cluster regulatory role in bovine adipocyte adipogenesis. In this study, miRNA agomir repressed adipogenesis (**A**) and miRNA antagomir increased adipogenesis (**B**). Since most of the proved target genes (*DCN*, *G6PD*, and *LPL*) were strong anti-adipogenic effects (solid blue lines), we hypothesize that there were other pro-adipogenic target genes apart from *GPAM* and *DGAT2* that contribute to *miR-23a~27a~24-2* cluster-regulated preadipocyte differentiation (dotted green lines).

As shown in Figure 6A,B, *miR-23a~27a~24-2* potentially regulated adipogenesis by regulating two types of targets: genes with anti-adipogenic effects (solid blue lines) and pro-adipogenic effects (dotted green lines). Because we verified that the *miR-23a~27a~24-2* cluster inhibits preadipocyte differentiation, the predominant role of the target genes should theoretically be pro-adipogenic. However, our results indicate that *DCN*, *G6PD* and *LPL* were all strongly anti-adipogenic in this study. It is possible that the effects of the two pro-adipogenic genes *GPAM* and *DGAT2* on adipogenesis were not strong enough to lead the inhibitory of adipogenesis when targeted by the *miR-23a~27a~24-2* cluster. Thus, we asked whether there were other pro-adipogenic target genes apart from *GPAM* and *DGAT2* that contribute to *miR-23a~27a~24-2* cluster-regulated preadipocyte differentiation. If this is true, the predominant pathway should be the pro-adipogenic (dotted green lines) pathway.

### 2.6. FGF11 Is a Direct Target of the miR-23a~27a~24-2 Cluster

**Figure 7:**
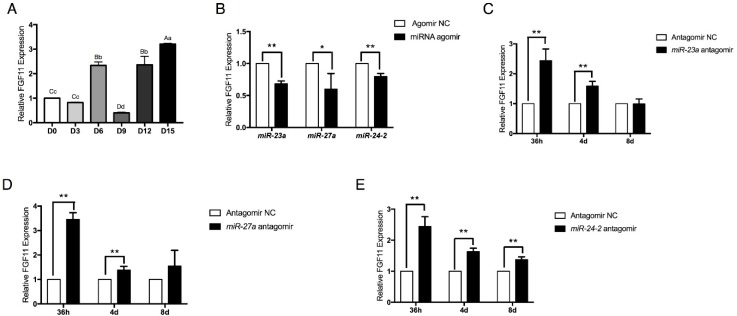
*FGF11* expression is commonly regulated by *miR-23a*, *miR-27a*, and *miR-24-2*. (**A**) Time course of *FGF11* expression during preadipocyte differentiation period. (**B**) *miR-23a* agomir, *miR-27a* agomir, and *miR-24-2* agomir suppressed *FGF11* expression in adipocytes. (**C**–**E**) Transfection of *miR-23a* antagomir, *miR-27a* antagomir, and *miR-24-2* antagomir increased *FGF11* expression in differentiating adipocytes from 36 h to 8 days. Error bars represent SEM. Lowercase represents *p* < 0.05. Uppercase represents *p* < 0.01. “*” represents *p* < 0.05. “**” represents *p* < 0.01.

To investigate whether there are other pro-adipogenic genes that are regulated by the *miR-23a~27a~24-2* cluster, predicted targets were further analyzed using TargetScan software and miRanda. Among the thousands of predicted genes, we identified a new gene that might be commonly targeted by *miR-23a*, *miR-27a*, and *miR-24-2*: *FGF11*. *FGF11* is an intracellular fibroblast growth factor and is a potential novel mediator of adipogenesis [32]. We found that expression of *FGF11* was clearly induced during adipocyte adipogenesis (Figure 7A). Furthermore, its expression pattern was opposite to that of *miR-27a* and *miR-24-2*. In addition, expression of *FGF11* was tightly controlled by the *miR-23a~27a~24-2* cluster. Agomir of *miR-23a*, *miR-27a*, and *miR-24-2* significantly repressed *FGF11* expression (Figure 7B). Conversely, inhibition of *miR-23a*, *miR-27a*, and *miR-24-2* strongly increased *FGF11* expression in adipocytes from 36 h to 8 days post transfection with miRNA antagomir (Figure 7C–E). These results indicate that *FGF11* is a potential target gene of the *miR-23a~27a~24-2* cluster.

**Figure 8:**
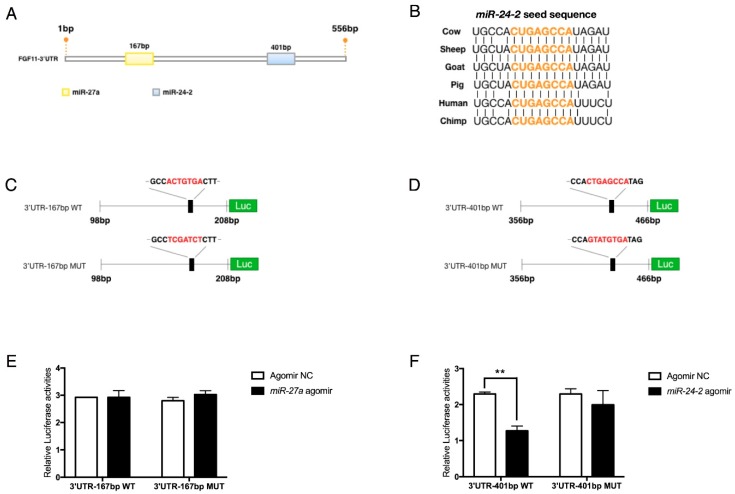
*FGF11* is targeted by the *miR-23a~27a~24-2* cluster. (**A**) Prediction of *miR-23a*, *miR-27a*, and *miR-24-2* seed sequence in the *FGF11* 3’UTR region using TargetScan software and miRanda. (**B**) Sequence alignments of *miR-24-2* seed sequence showed high conservation among mammals. (**C**,**D**) Structure of *FGF11*-3’UTR luciferase reporter vector with wild or mutant miRNA seed sequence. The mutant sequence was marked in red. (**E**,**F**) Luciferase analysis showed that *FGF11* was directly targeted by *miR-24-2* (F) but not *miR-27a* (**E**). Error bars represent SEM. “**” represents *p* < 0.01.

To determine the relationship between the *miR-23a~27a~24-2* cluster and *FGF11*, in vitro luciferase activity assays were performed in 293T cells. Two potential binding sites were predicted within the 3’UTR of *FGF11* mRNA (Figure 8A). The *miR-24-2* seed sequence between the two binding sites was highly conserved among mammals (Figure 8B). Next the wild-type and mutant 3’UTRs containing the miRNA binding site seed sequence were chemically synthesized and cloned into the psiCHECK™-2 vector (Figure 8C,D). Results showed that *miR-24-2* agomir significantly reduced luciferase activities of the *FGF11* 3’UTR reporter vector (Figure 8F) while *miR-27a* had no effects (Figure 8E). These results indicate that *FGF11* is commonly regulated by *miR-23a*, *miR-27a*, and *miR-24-2*, and is a direct target of *miR-24-2*.

### 2.7. FGF11 Is a Pro-Adipogenic Mediator of Adipogenesis

**Figure 9:**
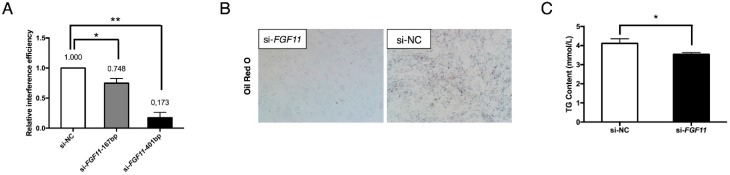
*FGF11* is a pro-adipogenic mediator of adipogenesis. (**A**) Interference efficiency of siRNA targeting *FGF11* mRNA in adipocytes. (**B**) Oil Red O staining of lipids in *FGF11* interfered differentiating adipocytes (magnification: 40×). (**C**) ELISA analysis of TG content in adipocytes when interfering *FGF11* expression by siRNA. Error bars represent SEM. “*” represents *p* < 0.05. “**” represents *p* < 0.01.

An siRNA interference assay was employed to investigate the effects of *FGF11* on adipocyte adipogenesis. Two *FGF11* siRNA oligonucleotides were designed and transfected into preadipocytes to inhibit *FGF11* expression. The siRNA with clearly higher interference efficiency (82.7%), si-*FGF11*-401bp, was finally chosen for the interference assays (Figure 9A). We found that inhibition of *FGF11* strongly reduced lipid droplet accumulation as visualized by Oil Red O staining (Figure 9B). ELISA analysis also showed that inhibition of *FGF11* obviously reduced cytoplasm TG content (Figure 9C). These results indicate that *FGF11* is a downstream pro-adipogenic target of the *miR-23a~27a~24-2* cluster. The co-regulation of *FGF11*, *GPAM*, and *DGAT2* might explain why preadipocyte differentiation is negatively regulated by the *miR-23a~27a~24-2* cluster.

## 3. Discussion

In beef cattle, fat deposition is closely associated with the meat quality of beef, which is one of the most important economic traits of beef production [33,34]. During adipogenesis, miRNA has been shown to be important for post-transcriptional regulation [35]. A previous study has demonstrated that in bovine subcutaneous adipose tissue, there are hundreds of miRNAs detectable with 17 miRNAs that are tissue-specifically expressed that might be involved in the regulation of energy balance [36]. Recently, it was reported that the predicted common targets of the *miR-23a~27a~24-2* cluster are enriched in energy metabolism-related pathways [12]. However, to our knowledge, the cooperative and independent functions of the *miR-23a~27a~24-2* cluster in bovine adipocyte adipogenesis has not been examined. 

Our study is the first to identify the cooperative functions of the miRNAs in the *miR-23a~27a~24-2* cluster in adipogenesis. Our principal findings are: (1) the miRNAs in the *miR-23a~27a~24-2* cluster inhibits bovine adipocyte adipogenesis; (2) the miRNAs in the *miR-23a~27a~24-2* cluster individually regulate *ATP5F1*, *DCN*, *GPAM*, *DGAT2*, *G6PD*, and *LPL* and cooperatively regulate *FGF11*; and (3) the inhibitory effect on adipogenesis is achieved through the balanced regulation by targeting two types of genes with pro- or anti-adipogenic effects.

Several studies have reported the roles of *miR-23a* and *miR-27a* in preadipocyte differentiation. *miR-23a* was identified as a negative regulator of preadipocyte differentiation by Huang et al. [18] and Guan et al. [19]. Many studies have demonstrated that *miR-27a* negatively regulates adipogenesis by targeting *PPARG*, the key transcription factor in adipocyte terminal differentiation [20,23,37,38]. In this study we confirmed these previously reported roles of *miR-23a* and *miR-27a*. We also showed that *miR-24-2* is a negative regulator of adipogenesis. Although both Huang et al. [18] and Guan et al. [19] measured miRNA expression during preadipocyte differentiation, conflicting expression patterns were reported. Huang et al. found that *miR-23a* expression first increased then decreased from day 2 to day 8 during 3T3-L1 preadipocyte differentiation [18]. However, Guan et al. reported that *miR-23a* was immediately down regulated during adipogenic differentiation of platelet derived growth factor receptor alpha (PDGFRα)-positive progenitor cells [19]. The expression pattern of *miR-23a* in our study was the same as that reported by Huang et al. As studies have reported that depot-specific differences in adipose tissue contribute differently to metabolism [1,29,39], we think that these differences in expression patterns are understandable and are largely due to the different cell models used.

Traditionally, miRNA is considered to post-transcriptionally repress expression by binding to the 3’UTR of mRNA [40,41]. In most cases, when the relationship between an individual miRNA and its target gene is studied, the miRNA and its target gene are found to have opposite effects [42,43,44]. However, because a single miRNA might bind to multiple mRNAs and multiple miRNAs might regulate a single mRNA [12], it is possible that these targets have opposite effects, even in the same biological process. Thus, regulation of these genes may be a way to maintain homeostasis. Interestingly, in this study, we identified three direct target genes (*DCN*, *G6PD*, and *LPL*) that have the same effects as what the miRNAs in the *miR-23a~27a~24-2* cluster do on adipocyte adipogenesis and three that have the opposite effects (*GPAM*, *DGAT2*, and *FGF11*).

We found that *miR-23a* targets *DCN*, *miR-24-2* targets *G6PD* and *LPL*, and that all these three genes inhibit adipogenesis. DCN is a recently identified secreted protein that inhibits preadipocyte differentiation [29]. Considering that it binds to a variety of collagens, it is likely that *DCN* inhibits adipogenesis through collagen-dependent pathways [45,46]. G6PD is a rate-limiting enzyme in the pentose phosphate pathway and is indispensable in de novo lipogenesis [24]. It has also been implicated in adipose tissue inflammation and systemic insulin resistance in obesity [27]. It was recently demonstrated that abnormal activation of G6PD increases cellular nicotinamide adenine dinucleotide phosphate (NADPH), resulting in lipid dysregulation in obesity [47]. However, here we found that *G6PD* clearly had the opposite effect on preadipocyte differentiation. This may be related to the locations of the fat depots where the cells were isolated [1]. LPL is a rate-limiting enzyme catalyzing the hydrolysis of TG and is synthesized and secreted by a variety of cell types including myocytes, adipocytes and mammary gland cells, implying that *miR-24-2* could inhibit adipocyte adipogenesis by repressing the hydrolysis of TG by *LPL* [30,48].

In the present study, we also identified three pro-adipogenic targets of the *miR-23a~27a~24-2* cluster: *GPAM* was targeted by *miR-27a*, *DGAT2* was targeted by *miR-24-2*, and *FGF11* was commonly regulated by *miR-23a*, *miR-27a*, and *miR-24-2*. GPAM, also known as glycerol 3-phosphate acyltransferase 1 (GPAT1), is one of four identified GPATs [49]. GPATs catalyze the first step of TAG synthesis and are rate-limiting enzymes in the de novo pathway of glycerolipid synthesis [50]. Studies have reported that mitochondrial *GPAM* greatly limits glycerolipid synthesis in bovine embryonic fibroblast cells [51]. Meanwhile, elevated *GPAM* expression level is related to higher bovine milk fat synthesis and increased adipose tissue lipogenesis [52,53]. DGAT2 is responsible for the final step of TAG synthesis: the conversion of diacylglycerols (DAGs) to TAG [54]. Thus, *DGAT2* might strongly contribute to *miR-23a~27a~24-2* cluster-regulated inhibition of adipogenesis. We also found that *FGF11* was commonly regulated via the *miR-23a~27a~24-2* cluster. FGF11 is an intracellular fibroblast growth factor that is involved in angiogenesis, tumorigenesis, and liver regeneration [55]. Importantly, a microarray study found that *FGF11* expression was dramatically increased during in vitro differentiation of human adipose-derived stem cells, indicating that *FGF11* might be a novel mediator of adipogenesis [32]. Consistent with this, we identified that *FGF11* is a positive regulator of preadipocyte differentiation. The contribution of the pro-adipogenic targets *GPAM*, *DGAT2*, and *FGF11* to adipogenesis might be greater than that of *DCN*, *G6PD*, and *LPL*, and this may lead to the reduced adipogenesis phenotype observed in the presence of the *miR-23a~27a~24-2* cluster. Although much more work is needed to elucidate the mechanisms underlying the cooperative functions of the *miR-23a~27a~24-2* cluster in adipogenesis, the newly identified co-regulatory roles in this study will also help inform future studies on adipogenesis.

In the studies of animal breeding, genomic selection is a powerful approach to improve the quality of economic traits [56]. The pivotal issue for genomic selection is to establish the reference population and candidate population for better selection of the paternal and maternal candidates [57]. To improve the meat quality of beef, increasing the marbling (or intramuscular fat) content is an efficient and necessary approach [58]. Understanding the molecular basis of the *miR-23a~27a~24-2* cluster in adipogenesis can provide new insights for selecting candidates with more intramuscular fat content. Consequently, the *miR-23a~27a~24-2* cluster can be considered as a candidate target for genomic selection breeding in beef cattle to improve the meat quality.

**Figure 10:**
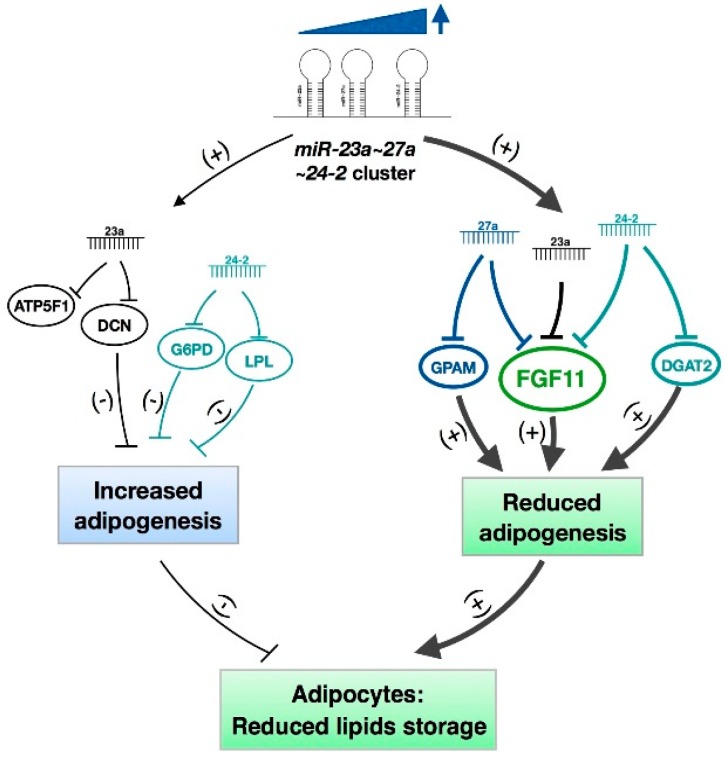
Mechanisms of the *miR-23a~27a~24-2* cluster in regulating bovine adipocyte adipogenesis. The *miR-23a~27a~24-2* cluster represses adipogenic differentiation of bovine preadipocytes. This inhibitory effect is achieved through a balanced regulation via targeting two types of genes with anti-adipogenic effects (*DCN*, *G6PD*, and *LPL*) and pro-adipogenic effects (*GPAM*, *DGAT2*, and *FGF11*), which finally leads to the *miR-23a~27a~24-2* cluster-mediated repression of adipogenesis.

In conclusion, the *miR-23a~27a~24-2* cluster represses adipogenic differentiation of bovine preadipocytes. This inhibitory effect is achieved through a balanced regulation via targeting two types of genes with anti-adipogenic effects (*DCN*, *G6PD*, and *LPL*) and pro-adipogenic effects (*GPAM*, *DGAT2*, and *FGF11*), which finally leads to the *miR-23a~27a~24-2* cluster-mediated repression of adipogenesis (Figure 10). Even though this study did not reveal the detailed mechanisms underlying the regulation of the *miR-23a~27a~24-2* cluster on adipogenesis, the newly identified cooperative and independent regulatory roles for the miRNAs in this cluster will provide insights into the mechanisms that govern how adipogenesis is regulated, and this knowledge can be used to increase the meat quality of beef.

## 4. Materials and Methods

### 4.1. Isolation of Bovine Preadipocytes and Cell Culture

Isolation and cell culture of preadipocytes were performed as previously described by Meissburger et al. and Wang et al. [29,59]. The experiments were performed in accordance with the guiding principles for the experimental animals adopted by Ministry of Science and Technology. Animal care was approved by the Institutional Animal Care and Use Committee of Northwest A&F University (approved code: No.142, 2005). The adipose sample was obtained from a slaughter house. The perirenal adipose tissue was removed, placed into phosphate buffer saline (PBS) supplemented with 10% penicillin/streptomycin (Hyclone, ThermoFisher Scientific, Shanghai, China) and immediately taken to the cell culture lab. The blood vessels and connective tissues were carefully dissected away from the adipose tissue. The adipose sample was then minced and digested with 0.25% collagenase I (Sigma, Shanghai, China) for 1 h at 37 °C in a shaking water bath. The cell suspension was filtered through a 40-μm cell strainer and centrifuged for 5 min at 350× *g* to obtain cell pellets. The pellets were resuspended and seeded in 60-mm collagen-coated cell culture plates. The preadipocytes were grown in growth medium containing Dulbecco’s Modified Eagle Medium/F-12 (DMEM/F-12, Gibco, Shanghai, China) supplemented with 10% fetal bovine serum (FBS, Gibco) and 1% penicillin/streptomycin (Gibco). Growth medium was changed every two days and cells were passaged at 70% confluence to avoid spontaneous differentiation. For adipogenic differentiation, post-confluent cells were cultured in induction medium containing DMEM/F-12 supplemented with 10% FBS, 1% penicillin/streptomycin, 5 μg/mL insulin (Sigma-Aldrich, Shanghai, China), 1 μM dexamethasone (Sigma-Aldrich) and 0.5 mM 3-isobutyl-1-methylxanthine (IBMX, Sigma-Aldrich) for 2 days. Then, every 2 days the cells were incubated in fresh maintenance medium containing DMEM/F-12 supplemented with 10% FBS, 1% penicillin/streptomycin, and 5 μg/mL insulin.

### 4.2. Oil Red O Staining

Lipid droplets were visualized using Oil Red O staining [60]. The Oil Red O stock solution was prepared by dissolving 0.5 g powder in isopropanol, and the solution was protected from light. The working solution was prepared by diluting the stock solution to 60% with sterile deionized water. To stain lipids, cells were washed with PBS, and fixed with 4% paraformaldehyde for 30 min at room temperature. After that, cells were rinsed with PBS three times and stained with Oil Red O working solution for 30 min at room temperature. The fluorescent dye 4’,6-diamidino-2-phenylindole (DAPI) was used to stain the cell nuclei at a concentration of 1.43 μM. Bright field and fluorescent microscopy were taken with an inverted fluorescence microscope (Olympus IX71, Olympus Corporation, Beijing, China).

### 4.3. RNA Oligonucleotides and Transfection

Chemically modified agomir and antagomir were used to increase and inhibit, respectively, the expression of *miR-23a*, *miR-27a*, and *miR-24-2*. The miRNA agomir and antagomir were synthesized and purified using high-performance liquid chromatography by GenePharma Co. Ltd (Shanghai, China). The sequences of the miRNA oligonucleotides are listed in Tables S1 and S2. The short interference RNA (siRNA) oligonucleotides targeting *DCN*, *GPAM*, *DGAT2*, *G6PD*, and *LPL* mRNAs and the negative control (NC) siRNA were designed using the online BLOCK-iT™ RNAi Designer (http://rnaidesigner.thermofisher.com/rnaiexpress). The siRNA oligonucleotides were chemically synthesized without modifications and purified using high-performance liquid chromatography by Sangon Biotech Co. Ltd (Shanghai, China). The sequences of the siRNA oligonucleotides are listed in Table S3.

For transfection of RNA oligonucleotides, cells were seeded in six-well cell culture plates in triplicate for each treatment. When the cells had grown to 70% confluence, miRNA agomir or siRNA oligonucleotides were transfected into cells using lipofectamine™ 3000 transfection reagent (Invitrogen, Shanghai, China) at a final concentration of 30 nM. miRNA antagomir was transfected at a final concentration of 75 nM according to the manufacturer’s protocol. Briefly, RNA oligonucleotides and transfection reagent were separately diluted in Opti-MEM medium (Gibco) and incubated for 10 min at room temperature. Next, the two mixtures were combined and incubated for another 15 min at room temperature to allow for the formation of transfection reagent-RNA complexes. The transfection complexes were then added to the cell culture medium drop by drop. Cells were incubated for 24 h before changing to a fresh growth medium.

### 4.4. Luciferase Activity Assay

To construct the luciferase reporter vector, ≈100–200 bp of the bovine 3′ untranslated region (3′UTR) region containing the wild type or mutant miRNA binding site was chemically synthesized (Sangon Biotech Co., Ltd.) and cloned into the psiCHECK™-2 (Promega, Beijing, China) vector. For co-transfection of miRNA oligonucleotides and the vector, 293A cells were seeded in 24-well cell culture plates in triplicate. For each treatment, 100 μg plasmid and 50 nM miRNA oligonucleotides were mixed and transfected into cells. The transfection protocol was the same as that used for miRNA and siRNA transfection.

The luciferase activity was measured 40 h after transfection using the Dual-Luciferase Reporter Assay System (Promega). In brief, cells were lysed in 1× Passive Lysis Buffer for 15 min at room temperature. To measure the firefly luciferase activity, 25 μL Luciferase Assay Buffer II was mixed with 10 μL cell lysate, and the absorbance was measured at 560 nm (TECAN, Infinite1 200 PRO NanoQuant, BioTek, Beijing, China). The Renilla luciferase activity was determined by adding 50 μL 1× Stop & Glo1 reagent to the cell lysate above and the absorbance was measured at 480 nm. The relative luciferase activity was calculated as the ratio of Renilla luciferase activity to firefly luciferase activity.

### 4.5. ELISA Analysis of Bovine Triglyceride Content

The TG content of the adipocytes was measured using the Bovine TG ELISA Kit (Jining Shiye, Shanghai, China). Briefly, equal number of cells were scraped into an equal volume of PBS and were repeatedly frozen and thawed to release the TG within the adipocyte cytoplasm. Cells were then centrifuged for 25 min at 2500 rpm to obtain the supernatant. The samples were then processed according to the manufacturer’s instructions. The final absorbance was measured at 450 nm (TECAN, Infinite1 200 PRO NanoQuant).

### 4.6. Quantitative Real-Time-PCR

Total RNA from the adipocytes (*n* = 3) was isolated using the RNAiso Plus kit (Takara Biomedical Technology Co., Ltd., Beijing, China) according to the manufacturer’s instructions. RNA was then reversely transcribed into cDNA using the Prime-Script™ RT reagent Kit with gDNA Eraser (Takara Biomedical Technology Co., Ltd.) for mRNA or the miRcute Plus miRNA First-Strand cDNA Synthesis Kit (TIANGEN, Beijing, China) for miRNA. The cDNA was then used as a template for qRT-PCR in triplicate wells in the 7500 Real Time PCR System (Applied Biosystems, Shanghai, China). TB Green™ Premix Ex Taq™ II (Tli RNaseH Plus, Takara, Beijing, China) was used for protein-coding gene analysis and the miRcute Plus miRNA qPCR Detection Kit (SYBR Green, TIANGEN) was used for miRNA analysis. The mRNA expression level was normalized to that of glyceraldehyde-3-phosphate dehydrogenase (*GAPDH*) and the miRNA expression level was normalized to that of U6. Three repeats of the qRT-PCR assays were performed. The experimental data were analyzed using the 2^−ΔΔ*C*t^ method [61]. Sequence information for the primers are provided in Table S4.

### 4.7. Statistical Analysis

All data were presented as the mean ± SEM. Statistically significant differences between two groups were tested using the independent-samples *t*-test, and significances among three or more groups were tested using one-way analysis of variance (ANOVA) [43].

## Supplementary Materials

Supplementary materials can be found at http://www.mdpi.com/1422-0067/19/12/3957/s1.

## Abbreviations

*GPAM*	glycerol-3-phosphate acyltransferase: mitochondrial
*DGAT2*	diacylglycerol O-acyltransferase 2
*DCN*	decorin
*G6PD*	glucose-6-phosphate dehydrogenase
*LPL*	lipoprotein lipase
*FGF11*	fibroblast growth factor 11
FBS	fetal bovine serum
IBMX	3-isobutyl-1-methylxanthine
siRNA	short interference RNA
NC	negative control
3′UTR	3′ untranslated region
TG	triglyceride
qRT-PCR	quantitative real time-PCR
ANOVA	one-way analysis of variance
*ATP5F1*	ATP synthase peripheral stalk-membrane subunit b
TAG	triacylglycerol
