# Supplementary material for: Cooperative and Independent Functions of the miR-23a~27a~24-2 Cluster in Bovine Adipocyte Adipogenesis
Source: Int J Mol Sci. 2018 Dec 9;19(12):3957. doi: 10.3390/ijms19123957 (PMC6321175; doi:10.3390/ijms19123957)
Supplement: Supplementary file 1 [file ijms-19-03957-s001.pdf]

Table S1. Information of the oligonucleotides of miRNA agomir.

RNA Oligo	Sense strand (5'-3')	Antisense strand (5'-3')	Purification	Modification
miR-23a agomir	AUCACAUUGCCAGGGAUUUCCA	GAAAUCCCUGGCAAUGUGAUUU	HPLC	2'-OMe
miR-27a agomir	UUCACAGUGGCUAAGUUCCG	GAACUUAGCCACUGUGAAUU	HPLC	2'-OMe
miR-24-2 agomir	UGGCUCAGUUCAGCAGGAACAG	GUUCCUGCUGAACUGAGCCAUU	HPLC	2'-OMe
Agomir NC	UUCUCCGAACGUGUCACGUTT	ACGUGACACGUUCGGAGAATT	HPLC	2'-OMe

Table S2. Information of the oligonucleotides of miRNA antagomir.

RNA Oligo	5'-3' sequence	Purification	Modification
miR-23a antagomir	UGGAAAUCCCUGGCAAUGUGAU	HPLC	2'-OMe
miR-27a antagomir	CGGAACUUAGCCACUGUGAA	HPLC	2'-OMe
miR-24-2 antagomir	CUGUUCCUGCUGAACUGAGCCA	HPLC	2'-OMe
Antagomir NC	CAGUACUUUUGUGUAGUACAA	HPLC	2'-OMe

Table S3. Information of the oligonucleotides of siRNA.

RNA Oligo	Sense strand (5'-3')	Antisense strand (5'-3')	Purification
si-DCN	GCUGAAGAGCUCAGGCAUUTT	AAUGCCUGAGCUCUUCAGCTT	HPLC
si-GPAM	GCUAGCAAGUCCUGUGCUATT	UAGCACAGGACUUGCUAGCTT	HPLC
si-DGAT2	GCAAGAAGUUCCCUGGCAUTT	AUGCCAGGGAACUUCUUGCTT	HPLC
si-G6PD	CCUGCGUCAUUCUCACCUUTT	AAGGUGAGAAUGACGCAGGTT	HPLC
si-LPL	CCACGAACGUUCCGUUCAUTT	AUGAACGGAACGUUCGUGGTT	HPLC
si-FGF11-267bp	GCUUUAAGGAGUGCGUCUUTT	AAGACGCACUCCUUAAAGCTT	HPLC
si-FGF11-401bp	GGAAAUGGUCCUGGCUGAUTT	AUCAGCCAGGACCAUUUCCTT	HPLC
si-NC	GGAGUUGCCGUCGUAAGAUTT	AUCUUACGACGGCAACUCCTT	HPLC

Table S4. Summary information of the genes used for qRT-PCR in this study.

Gene	GenBank Transcript ID	Purpose	Forward primer sequence (5′-3′)	Reverse primer sequence (5′-3′)	Product size
GAPDH	NM_001034034 (*Bos taurus*)	Gene expression	AGTTCAACGGCACAGTCAAGG	ACCACATACTCAGCACCAGCA	124bp
PPARG	NM_181024 (*Bos taurus*)	Gene expression	GACCACTCCCATGCCTTTGA	AACCATCGGGTCAGCTCTTG	108bp
CEBPA	NM_176784 (*Bos taurus*)	Gene expression	GCCTTCAACGACGAGTTCCT	CGGGTAGTCAAAGTCGTTGC	108bp
SREBP1	NM_001113302 (*Bos taurus*)	Gene expression	CGAGCCACCCTTCAACGAA	AGCATGTCTTCTATGTCGGTCA	93bp
ATP5F1	NM_001038501 (*Bos taurus*)	Gene expression	TGTAACAGGACCCTATGTGCTTG	TCCCCAACAGAAGCACCATA	150bp
DCN	NM_173906 (*Bos taurus*)	Gene expression	TCGTCGTAGAACTTGGCACC	CAGCAATGCGGATGTAGGAG	96bp
GPAM	NM_001012282 (*Bos taurus*)	Gene expression	AGCATTGGTCGGTGTAAGCA	AGACGGGATACTGGGGTTGA	189bp
DGAT2	NM_205793 (*Bos taurus*)	Gene expression	GCTCCAAGTCATCTCGGTGCT	GACCTCCTGCCACCTTTCTT	171bp
G6PD	NM_001244135 (*Bos taurus*)	Gene expression	CTTTGGCACTGAGGGTCGT	GCCTGCACCTCTGAGATACACT	180bp
LPL	NM_001075120 (*Bos taurus*)	Gene expression	GATGATGCGGATTTTGTAGACG	GTTGGAAAGTGCCTCCGTTAG	121bp
FGF11	NM_001192939 (*Bos taurus*)	Gene expression	GAACCTCAGCTCAAAGGCATC	GGTTGAAGTGGGTGAAAGAGC	124bp
